# Associations between rural/urban status, duration of untreated psychosis and mode of onset of psychosis: a mental health electronic clinical records analysis in the East of England, UK

**DOI:** 10.1007/s00127-024-02758-3

**Published:** 2024-09-09

**Authors:** Karolina Kaminska, Jo Hodgekins, Jonathan R. Lewis, Rudolf N. Cardinal, Sherifat Oduola

**Affiliations:** 1 Norwich Medical School, University of East Anglia, Norwich Research Park, Norwich, NR4 7TJ UK; 2 Department of Psychiatry, University of Cambridge, Clifford Allbutt Building, Bay 13, Cambridge Biomedical Campus, Cambridge, CB2 0AH UK; 3 Cambridgeshire and Peterborough NHS Foundation Trust, Fulbourn Hospital, Elizabeth House, Fulbourn, Cambridge, CB21 5EF UK; 4 School of Health Sciences, University of East Anglia, Norwich Research Park, Norwich, NR4 7TJ UK

**Keywords:** First-episode psychosis, Duration of untreated psychosis, Rural, Urban, Sociodemographic characteristics

## Abstract

**Purpose:**

The influence of rurality on the duration of untreated psychosis (DUP) in first-episode psychosis (FEP) is poorly understood. We investigated factors associated with FEP in rural/urban settings and whether there are rural/urban differences in DUP and the mode (speed) of onset of psychosis.

**Methods:**

We used the Cambridgeshire and Peterborough NHS Foundation Trust Research Database (CPFTRD) to identify all persons presenting to an early intervention for psychosis service with FEP between 2013 and 2015. We performed descriptive statistics and multivariable linear and multinomial regression to assess the relationships between the study outcomes and the independent variables.

**Results:**

One hundred and fifty-five FEP patients were identified, with a mean age of 23.4 (SD, 5.3) years. The median DUP was 129.0 (IQR: 27.5–524.0) days. In rural areas, FEP patients were more likely to be employed and live with family than those in urban areas. A longer DUP was observed among patients with an insidious onset of psychosis compared with an acute onset (619.5 (IQR: 333.5–945.0)) vs. (17.0 (IQR: 8.0–30.5)) days respectively, *p* < 0.0001. We found evidence that the mode of onset of psychosis differed by employment status and living circumstances. There was insufficient evidence of rural/urban differences in DUP and mode of onset of psychosis.

**Conclusions:**

Our results suggest that the mode of onset of psychosis is an important indicator of treatment delay and could provide vital information for service planning and delivery. Sociodemographic variations in FEP exist in rural populations, and our findings are similar to those observed in urban settings.

**Supplementary Information:**

The online version contains supplementary material available at 10.1007/s00127-024-02758-3.

## Introduction

Duration of untreated psychosis (DUP), usually defined as the time from the onset of frank psychotic symptoms (i.e., hallucinations or delusions) to the date of first contact with a mental health service for psychosis or the start of antipsychotic treatment, is an important indicator of illness prognosis. A prolonged DUP is associated with poor clinical outcomes [1, 2], reduced social functioning and poor quality of life [2, 3]. Previous research has linked individual, service-related and environmental factors to DUP [4–6], but this has often been gleaned from the urban population perspectives. Few studies in low-to-middle-income countries have considered the rural/urban effect of DUP [7, 8], but the findings are heterogeneous. In the UK context, there is a severe lack of research on the relationships between DUP and rurality. Understanding the factors and processes contributing to treatment delays is important to strengthen the chances of recovery for people living in rural areas with first episode psychosis (FEP).

Several sociodemographic factors are associated with DUP, including social support, ethnicity, living circumstances, age, and employment status [9–12]. Establishing whether these factors are also important in rural contexts is essential. The link between neighbourhood factors such as urbanicity, deprivation, substance misuse and geographical accessibility to psychiatric services and DUP has been studied [4, 6, 13]. In a cross-country study of DUP in Mexico and the USA, Fresan et al. (2020) showed similarities in the median DUP in both countries (35 and 38 weeks, respectively) [14].

In the UK, national attention is being paid to the multiple barriers that people living in rural and remote areas face in accessing timely treatment for mental health difficulties, and the Chief Medical Officer calls for urgent actions to reduce rural health inequalities [15]. The barriers include geographic isolation, reduced access (e.g., transportation) to services and lower socioeconomic status [16, 17]. Further, the impact of shame, stigma and lack of awareness about the signs and symptoms of psychosis is also considerable and could influence DUP [18]. It is well documented that mental illnesses’ stigma can be more prevalent in rural areas, leading to a reluctance to seek treatment [19, 20]. This is particularly concerning, as research has shown that stigma can be a significant barrier to seeking help for psychosis [21, 22].

The onset of first episode psychosis is often preceded by a prodromal phase which is characterised by reduced functioning and subtle symptoms [23]. Understanding this early phase of psychosis could potentially provide possible mechanisms for improving the course of the illness. For example, intervening at the start of symptoms, improving mental state and access to mental health treatment [24] could halt the development of FEP and reduce DUP. When examined by the prodromal phase, DUP displays a complex pattern. The mode of onset of psychosis is defined as the speed at which psychotic symptoms develop, including an acute onset (within days or a week) or in a more gradual way, for more than a few months [25]. Indeed, the association between the mode of onset of psychosis and DUP has been studied [25, 26]. An acute mode of onset of psychosis is found to be associated with shorter DUP [25, 27]. Patients who experienced gradual/ insidious onset of symptoms have been reported to be less likely to seek help immediately or because they have a poor insight into their illness. An insidious or gradual onset of psychosis could also prevent significant others to seek help on behalf of the patient; this could be because an insidious onset is more difficult to recognise, which might become a barrier to help-seeking [28, 29]. The extent to which these findings could be replicated in rural populations is unknown.

Considering dearth of research on the relationships between DUP, mode of onset of psychosis and among the rural populations in the UK, we assembled an epidemiologically characterised sample of first episode psychosis patients to examine these issues. Our aims were to investigate: (a) whether the characteristics of FEP patients differed by rural/urban status; (b) whether DUP and mode of onset of psychosis differed by sociodemographic characteristics, and (c) whether rural/urban differences in DUP and mode of onset of psychosis exist or remained, after controlling for confounders.

## Methods

### Samples

The study sample was drawn from Cambridgeshire and Peterborough in the East of England region of the UK. Geographically, the catchment areas consist of rural and urban locations with a population of ~ 0.9 million people [30]. Cambridgeshire and Peterborough NHS Foundation Trust (CPFT) is the sole secondary care mental health provider serving the population.

### Study design, data source, and participants

We used data from an ongoing longitudinal incidence study of first episode psychosis in Cambridgeshire and Peterborough. Using the Cambridgeshire and Peterborough NHS Foundation Trust Research Database (CPFTRD), we identified all persons presenting with a first episode psychosis (World Health Organization [WHO] International Classification of Diseases, Tenth Revision [ICD-10] codes F20 to F29) [31] who presented to an early intervention for psychosis service (EIS) in CPFT between 2013 and 2015. The CPFTRD is a de-identified copy of CPFT electronic clinical records [32] and contains patient records from hospital and community services. The relevant CPFT electronic clinical records system (RiO) became operational in 2013. Data within CPFTRD are available in two formats: (a) structured fields (e.g., demographic, diagnosis information) and (b) unstructured fields (i.e., free text). We searched the CPFTRD for demographic and clinical information and identified all potentially eligible participants.

### Procedure

Our case ascertainment procedures were modelled on those used in the Clinical Records Interactive Search-First Episode Psychosis (CRIS-FEP) study [9, 33]. First, we used Structured Query Language (SQL) [34] to interrogate the structured and free-text fields in CPFTRD to retrieve the records of patients presenting to an EIS between 2013 and 2015 (see Supplementary Material 1); then we applied defined search terms (e.g.,‘psychos*’; ‘onset’; ‘psychosis’; ‘voices’). This returned records of probable participants. Second, the research team screened each patient’s de-identified records for eligibility using the Screening Schedule for Psychosis [35] and the study inclusion/ exclusion criteria. Third, the research team reviewed the de-identified clinical records of the eligible participants to determine their DUP (i.e., the date of onset of psychosis and first contact with CPFT) and mode of onset and extract the study variables. Two researchers (KK and SO) independently extracted data on DUP, and an interrater reliability test was performed between the two researchers on a random 15% of the sample (*n* = 20). A kappa score of 0.75, *p* < 0.001 was achieved, indicating a substantial agreement. Discrepant or ambiguous cases were resolved by consensus within the research team.

### Inclusion/exclusion criteria

The inclusion and exclusion criteria were based on those used in the CRIS-FEP study [33]. Participants were included if they were (a) resident in Cambridgeshire and Peterborough areas between May 2013 and April 2015, (b) were accepted by an EIS between these times (c) were diagnosed with a psychotic disorder (ICD F20-29), and (d) were 14 to 35 years old at first presentation for psychosis. Exclusion criteria were (a) evidence of psychotic symptoms being due to an acute intoxication, b) and/or those being due to organic illness; c) evidence of previous contact with services for psychotic symptoms.

### Outcome variables and measures

#### DUP

Data relating to date of onset of psychosis were collected in CPFTRD using the Personal and Psychiatric History Schedule (PPHS) [36]. DUP was defined as the period in days from the date of onset of psychotic symptoms to the date of first contact with CPFT for psychosis. In line with previous studies [9, 37], onset of psychosis was defined as the presence for one day or more of one of the following psychotic symptoms: delusions, hallucinations, marked thought disorder, marked psychomotor disorder, and bizarre, grossly inappropriate and/or disorganized behaviour etc. Our end point for DUP was contact with early intervention service in CPFT. For regression analysis purposes, we used log transformation to estimate the coefficients of DUP by rural/urban status, since DUP was positively skewed.

#### Mode of onset of psychosis

Mode of onset of psychosis is defined as the speed at which psychotic symptoms develop, such as with an acute onset (within days or a week) or in a more gradual way, across several months [25]. Mode of onset data using the PPHS [36] and initially classified according to five categories: abrupt (within hours/ days), acute (within one week), moderately acute (within one month), gradual (within six months) and insidious (more than six months). The PPHS is a schedule previously used in the WHO multi-centre studies of the incidence and outcome of schizophrenia [35] and has been used in other landmark studies such as AESOP [37]. It has been shown to be psychometrically sound with good validity and reliability. For statistical analysis and due to the small sample, we collapsed mode of onset into three categories as follows: Acute (encompassing abrupt/ acute/ moderately acute), Gradual (gradual) and Insidious (insidious). These re-categorisations have been used in previous studies [9, 11].

### Covariates and measures

#### Sociodemographic variables

The Medical Research Council Sociodemographic Schedule [38] was used to collect data on sociodemographic characteristics, i.e., gender, age, ethnicity, education, living circumstances, relationship, and employment status. Ethnicity was categorised according to the 18 categories of the 2011 UK Census [39]. Due to small numbers in each category, for analysis purposes and in keeping with previous studies [25, 40], we collapsed ethnicity into five categories as follows: white British, black African/Caribbean and mixed (black African, black Caribbean, other black, mixed); Asian (other Indian, Pakistani, Bangladeshi, Chinese); other (Arab, any other ethnic group) white non-British (white Irish, white Gypsy, white Other). For analysis, we categorised age in to three groups: 14–17 years; 18–25 years and 26–35 years. These reflect previous research on the developmental presentation studies suggest adolescence is associated with a long DUP [41, 42], whilst others show that young adults (18–25 years) have a shorter DUP [43, 44].

#### Rural/urban status

In the CPFTRD, patients’ residential addresses (including, e.g., postcodes) are removed but replaced with the UK Office for National Statistics (ONS) administrative geographical level of Lower layer Super Output Area (LSOA) information. The ONS Rural-Urban Classifications linked to LSOA were used to determine patients’ rural/urban status [45]. The ONS Rural-Urban Classification assigns areas to one of four urban categories (major conurbation; minor conurbation; city and town; city or town in sparse settings) or six rural categories (town or fringe; town or fringe in sparse settings; village; village in sparse settings; hamlets and isolated dwellings; hamlets and isolated dwellings in a sparse setting) [45]. These categories were then collapsed into two: urban and rural, in line with the ONS guidelines [45].

### Statistical analysis

Data were analysed in RStudio 4.0.3 [46]. Results are reported following the RECORD checklist (see Supplementary Material 2) for routinely collected health data studies [47]. Descriptive statistics, including frequencies, percentages, for categorical data, means, and medians, along with the standard deviation and interquartile range for continuous data were used to describe the sample. We performed chi-square (or Fisher exact tests as appropriate) and t tests to (a) compare sociodemographic and clinical characteristics between rural and urban FEP patients, (b) examine associations between mode of onset of psychosis and sociodemographic and rural/urban characteristics. DUP was heavily skewed and was consequently log-transformed to allow parametric analyses. DUP for each group of patients is presented in the original scale, while the linear regression analyses were conducted using the logarithmic-transformed values. The Kruskal Wallis test was employed to estimate differences in DUP by sociodemographic, clinical, and rural and urban characteristics. Missing data were handled via multiple imputation by fully conditional specification using chained equations [48]. To minimise the risk of Type 1 error, the Bonferroni method [49] was used to adjust the p-values for the study outcomes due to the multiple comparisons in the inferential statistics. Finally, to examine rural/urban differences in DUP and mode of onset of psychosis, we fitted crude and multivariable linear and multinomial logistic regression models, controlling for a-priori confounders (age, gender, ethnicity), then adjusted for variables associated with the dependent variables (employment status and living circumstances). We defined statistical significance as *p* < 0.05 in the descriptive statistics and reported odds ratios along with 95% confidence intervals in the regression models. Aside from the descriptive statistics reported in Table 1, all other analyses were conducted with imputed data.

**Table 1:** Sample characteristics

Variable	*N* = 155 (%)
**Gender**	
Male	110 (71.0)
Female	45 (29.0)
**Mean Age (SD) years**	23.4 (5.3)
**Median DUP (IQR)**	129 (27.5–524.0)
**Ethnicity** ^**a**^	
White British	82 (56.2)
Black African/Caribbean	12 (8.2)
White non-British	26 (17.8)
Asian	13 (8.9)
Other	13 (8.9)
**Education** ^**b**^	
School, no qualification	25 (17.6)
School with qualification	45 (31.7)
Tertiary	51 (35.9)
Higher	21(14.8)
**Living circumstances** ^**c**^	
Alone	16 (10.3)
Family/relatives	109 (70.3)
Other	30 (19.4)
**Employment status** ^**d**^	
Employed	54 (34.8)
Student	38 (24.5)
Unemployed	60 (38.7)
**Relationship status** ^**e**^	
In a relationship	44 (28.9)
Single	108 (71.7)
**Rural/ urban status**	
Rural	46 (29.7)
Urban	109 (70.3)
**Mode of onset**	
Acute	55 (35.5)
Gradual	36 (23.2)
Insidious	64 (41.3)

Missing data: a = 9 patients; b = 13 patients; c = 13 patients; d = 3 patients; e = 3 patients

IQR: interquartile range; SD: standard deviation

### Ethical approval

The CPFTRD was approved by an NHS Research Ethics Committee (reference: 17/EE/0442) for secondary analysis. This study received Health Research Authority approval (reference: 20/NI/0035) and local CPFTRD Oversight Committee approval (reference: M00964) was obtained. Under UK law, patient consent was not required for this study.

## Results

### Sample characteristics

Two hundred and twenty-four patients presented to an EIS in CPFT between 2013 and 2015. Of these, 69 people were excluded as follows: 66 due to previous history of psychosis, 2 did not present with a psychotic disorder, and one patient had psychotic symptoms due to an organic illness. A total of 155 FEP patients met the study inclusion criteria. Table 1 describes the study sample. The mean age was 23.4 [standard deviation (sd), 5.3] years, there were more men (71.0%), and the majority were of white British ethnic group (56.2%), while 8.2% were of black African-Caribbean ethnic group. The median DUP was 129.0 (interquartile range (IQR): 27.5–524.0) days. An insidious mode of onset of psychosis was observed in many of the patients (41.3%).

### Rural/urban differences in patients with first episode psychosis

Table 2 shows rural/urban differences. Compared with patients in urban areas, FEP patients in rural areas, were more likely to live with family/relatives (rural: 84.8% vs. urban: 64.2%, *p* < 0.001); and were more likely to be employed (rural: 47.8% vs. urban: 29.3%, *p* < 0.001). The strength of these associations held after correcting the p-values. In both rural and urban areas, most patients were of white British ethnic group (63.0% and 54.1%, respectively). We observed that a small proportion of patients in rural settings were of black African/Caribbean (4.3%) or Asian (6.5%) ethnic groups, compared with the white British group (63.0%). There were no rural/urban differences by the mode of onset of psychosis, age, gender, or relationship status.

**Table 2:** Rural/urban differences in patients with first episode psychosis

	Rural *n* = 46 (%)	Urban *n* = 109 (%)	X^2^ /t tests (df), *p*	Bonferroni corrected *p*
**Gender**			2.23 (1), 0.12	0.11
Male	37 (80.4)	73 (67.0)		
Female	9 (19.6)	36 (33.0)		
**Mean Age (SD) years**	22.8 (5.7)	23.6 (5.2)	t = − 0.073 (77.9), 0.43	0.45
**Ethnicity**			2.24 (4), 0.74	0.75
White British	29 (63.0)	59 (54.1)		
Black African/Caribbean	2 (4.3)	11 (10.1)		
White non-British	8 (17.4)	19 (17.4)		
Asian	3 (6.5)	10 (9.2)		
Other	4 (8.7)	10 (9.2)		
**Education**			3.36 (3), 0.36	0.37
School, no qualification	6 (13.0)	23 (21.1)		
School with qualification	17 (37.0)	30 (27.5)		
Tertiary	17 (37.0)	38 (34.9)		
Higher	6 (13.0)	18 (16.5)		
**Living circumstances**			**7.67 (2)**,** 0.001**	**< 0.001**
Alone	4 (8.9)	12 (11.0)		
Family/relatives	39 (84.8)	70 (64.2)		
Other	3 (6.5)	27 (24.8)		
**Employment status**			**8.63 (2)**,** 0.001**	**< 0.001**
Employed	22 (47.8)	32 (29.3)		
Student	12 (26.1)	27 (24.8)		
Unemployed	12 (26.1)	50 (45.9)		
**Relationship status**			1.80 (1), 0.17	0.26
Single	29 (63.0)	82 (75.2)		
In a relationship	17 (37.0)	27 (24.8)		
**Mode of onset**			0.77 (2), 0.67	1.00
Acute	14 (30.4)	41 (37.6)		
Gradual	12 (26.1)	24 (22.0)		
Insidious	20 (43.5)	44 (40.4)		

X^2^ = chi-sq. test; df = degree-of-freedom; SD = standard deviation

### Associations between duration of untreated psychosis, sociodemographic characteristics

There was strong evidence of an association between DUP and mode of onset of psychosis. The longest median DUP was observed among patients with an insidious onset of psychosis compared with those with an acute onset [619.5 (IQR: 333.5–945.0)] vs. [17.0 (IQR: 8.0–30.5)] days respectively, *p* < 0.0001, (Table 3). There was weak evidence that patients aged 14–17 years old [326.0 (IQR: 60.0–703.0] experienced a longer median DUP compared with those aged 18–25 years old [101.0 (21.5–413.0), *p* = 0.08]. Although DUP did not differ statistically by sociodemographic characteristics, it is noteworthy that a longer median DUP was observed among the rural (131.5 days) patients compared with their urban (115.0 days) counterparts. Further, compared with white British patients (103.0 days), there was a trend that those of Asian (184.0 days) and ‘other’ (167.0 days) ethnic groups had a longer median DUP. Unemployed patients (161.5 days) also experienced a longer median DUP compared with those in employment (74.0 days). Neither of these differences reached statistical significance, (see Table 3).

**Table 3:** Associations between duration of untreated psychosis and sociodemographic characteristics

	Number in sample	Median (IQR) days	Kruskal-Wallis’ test (df), *p*	Bonferroni corrected *p*
**Gender**			1.23 (1), 0.26	0.27
Male	110	158.0 (33.2 -531.5)		
Female	45	58.0 (22.0–376.0)		
**Age-band**			4.88 (2), 0.09	0.08
14–17 years	23	326.0 (60.0–703.0)		
18–25 years	77	101.0 (21.5–413.0)		
26–35 years	55	132.0 (22.0–509.0)		
**Ethnicity**			2.53 (4), 0.63	0.67
White British	88	103.0 (29.7–5195)		
Black African/Caribbean	13	137.5 (16.5–231.0)		
White non-British	27	100.5 (19.5–480.5)		
Asian	13	184.0 (129.0–591.0)		
Other	14	167.0 (102.0–666.0)		
**Education**			3.53 (3), 0.31	0.39
School, no qualification	29	192.0 (37.0–539.0)		
School with qualification	47	144.0 (37.0–415.0)		
Tertiary	55	101.0 (30.5–547.0)		
Higher	24	84.0 (19.0–225.0)		
**Living circumstances**			2.59 (2), 0.27	0.47
Alone	16	148.5 (76.5–701.5)		
Family/relatives	109	142.0 (32.0–474.0)		
Other	30	79.0 (10.7–510.5)		
**Employment status**			2.86 (2), 0.23	
Employed	54	74.0 (14.0–592.7)		
Student	39	98.5 (33.5–498.2)		
Unemployed	62	161.5 (63.2–464.2)		
**Relationship status**			0.23 (1), 0.63	0.52
In a relationship	44	114.0 (24.7–344.5)		
Single	111	137.0 (31.2–582.7)		
**Rural/ urban status**			0.61 (1), 0.43	0.43
Rural	46	131.5 (40.7–647.5)		
Urban	109	115.0 (22.0–461.0)		
**Mode of onset**			**127.29 (2)**,** < 0.001**	**< 0.001**
Acute	55	17.0 (8.0–30.5)		
Gradual	36	103.5 (83.0–142.5)		
Insidious	64	616.5 (333.5–945.0)		

X^2^ = chi-sq. test; df = degree-of-freedom; IQR = interquartile range; SD = standard deviation

### Associations between mode of onset of psychosis and sociodemographic characteristics

Employment status was strongly associated with the mode of onset of psychosis (Table 4). We observed that patients with an acute onset of psychosis were more likely to be employed compared to those with a gradual or insidious onset (acute: 47.3% vs. gradual: 16.7%; acute: 47.3% vs. insidious: 37.5%, *p* = 0.02). In terms of living circumstances, high proportions of patients with an acute, gradual, or insidious onset lived with family/relatives. However, patients with an acute onset of psychosis were less likely to live alone than those with a gradual or insidious onset (acute: 5.4% vs. gradual: 16.7%; acute: 5.4% vs. insidious: 10.9, *p* = 0.04), see Table 4. These differences held after the p-value adjustments. There was no evidence of an association between the mode of onset and other sociodemographic characteristics or rural/urban status.

**Table 4:** Associations between mode of onset of psychosis and sociodemographic characteristics

	Acute *n* = 55 (%)	Gradual *n* = 36 (%)	Insidious *n* = 64 (%)	X^2^ / t tests (df), *p*	Bonferroni corrected *p*
**Gender**				2.43 (1), 0.31	0.39
Male	35 (63.6)	26 (72.2)	49 (76.6)		
Female	20 (36.4)	10 (27.8)	15 (23.4)		
**Mean Age (SD) years**	23.2 (5.1)	23.6 (5.4)	23.4 (5.5)	t = 0.04 (2), 0.95	0.96
**Ethnicity**				8.87 (4), 0.35	0.36
White British	34 (61.8)	20 (55.5)	35 (54.7)		
Black African/Caribbean	5 (9.1)	5 (13.9)	3 (4.7)		
White non-British	11 (20.0)	4 (11.1)	11 (17.2)		
Asian	3 (5.5)	2 (5.6)	8 (12.5)		
Other	2 (3.6)	5 (13.9)	7 (10.9)		
**Education**				6.78 (3), 0.36	0.37
School, no qualification	9 (16.4)	6 (16.7)	15 (23.4)		
School with qualification	15 (27.3)	11 (30.6)	22 (34.3)		
Tertiary	18 (32.7)	16 (44.4)	17 (26.6)		
Higher	13 (23.6)	3 (8.3)	10 (15.6)		
**Living circumstances**				**5.09 (2)**,** 0.05**	**0.04**
Alone	3 (5.4)	6 (16.7)	7 (10.9)		
Family/relatives	28 (69.1)	23 (63.9)	48 (75.0)		
Other	14 (25.5)	7 (19.4)	9 (14.1)		
**Employment status**				**9.97 (2)**,** 0.03**	**0.02**
Employed	26 (47.3)	6 (16.7)	24 (37.5)		
Student	13 (23.6)	12 (33.3)	13 (20.3)		
Unemployed	16 (29.1)	18 (50.0)	27 (42.2)		
**Relationship status**				2.44 (1), 0.31	0.36
Single	36 (65.5)	29 (80.6)	46 (71.9)		
In a relationship	19 (34.5)	7 (19.4)	18 (28.1)		
**Rural/ urban status**				0.77 (1), 0.67	0.67
Rural	14 (25.5)	12 (33.3)	20 (31.3)		
Urban	41 (74.5)	24 (66.7)	44 (68.7)		

Note: Bold estimates are statistically significant

### Rural/urban differences in DUP and mode of onset of psychosis

The crude and multivariable linear regression analysis revealed there was no evidence of rural/urban differences in DUP unadjusted β = 0.29 (95% CI: -0.32–0.91); adjusted β = 0.27 (95% CI: -0.39–0.95). Similarly, in our multinomial logistic regression analysis, we found insufficient evidence that patients from rural areas were more likely to experience a gradual (adjusted OR = 2.14; 95% CI: 0.73–6.27) or insidious (adjusted OR = 1.25; 95% CI; 0.51–3.04) onset of psychosis compared with patients living in urban areas, (see Table 5).

**Table 5:** Associations between mode of onset of psychosis and rural/urban status, analysed using multinomial logistic regression

	Gradual		Insidious	
	Unadjusted OR (95%CI)	Adjusted OR † (95%CI)	Unadjusted OR (95%CI)	Adjusted OR † (95%CI)
Urban	1.00	1.00		
Rural	1.46 (0.58–3.67)	2.14 (0.73–6.27)	1.33 (0.59–2.97)	1.25 (0.51–3.04)

**Mode of onset reference category: ** Acute

†Adjusted for age, gender, ethnicity, employment status and living circumstances

CI = confidence interval; OR = odds ratio

## Discussion

### Main findings

We conducted an incidence study of first episode psychosis to examine the relationships between DUP, mode of onset of psychosis, rural/urban status, and sociodemographic characteristics. Our findings suggest that patients living in rural areas tended to be more connected with family/relatives and more likely to be employed. Conversely, urban patients in our sample were more likely to be unemployed. Despite the lack of difference in DUP and mode of onset of psychosis by rural/urban status, we found strong evidence that patients with an insidious mode of onset of psychosis experienced a longer DUP. Living circumstances and employment status were also strongly linked to the mode of onset of psychosis, with our findings indicating that those in employment and living with family/relatives were more likely to experience an acute mode of onset of psychosis.

### Methodological considerations

This is one of a handful of studies in the global North that have investigated the influence of rurality on DUP and the mode of onset of psychosis, particularly in the UK. Our case identification and inclusion/exclusion criteria were based on a previous case register study [9, 33]. We reviewed the de-identified electronic health records of every potential FEP patient carefully to determine DUP, in line with previous studies [9, 37]. This gives a clearer picture of treatment delays in people presenting with psychosis for the first time to a large regional mental health provider serving both urban and rural populations totalling approximately 0.9 million people. Another strength is the use of multiple imputations to address missing data.

Our study has some limitations to consider when interpreting the results. First, future research with larger sample sizes exploring the onset of psychosis and DUP in rural populations is needed. Another potential limitation is in the collapsing of the ethnicity variable, meaning that our minority ethnic groups were heterogeneous. Additionally, even though we used a standardised instrument to measure DUP, the data on the onset of psychotic symptoms and first access to the secondary mental health service were extracted from the clinical records; therefore, the quality of the data was based on the robustness of clinical documentation. Furthermore, given our data source is limited to a small region of the UK, our findings may not be generalizable to other areas serving different populations. However, we note and discuss similarities in our results with other international studies in rural areas, see below. Precise measurement of DUP is considered difficult in the FEP population even when patients/participants are interviewed, this being often prone to recall bias, leading to variations in estimates. Similarly, selection bias may have affected our findings; despite our careful dating of DUP, our measurement relied only on secondary care data. Therefore, information about the onset of psychosis held by other services, e.g., non-statutory health providers, may have been missed, as these are not routinely available in secondary care records. Future research could consider overcoming these issues through data linkage to other nationally representative data sources.

### Interpretations of findings and relationship to previous studies

Only a few international studies have examined the relationships between DUP and rural/urban status. Our findings of no relationship between DUP and rural/urban status are consistent with findings by Thirthalli et al. (2017), who examined rural/urban differences in treatment-seeking among patients with psychosis in an Indian sample of 551 patients and found no differences in the duration of untreated illness between rural and urban patients [50]. Further, our findings that a greater proportion of rural patients live with family/relatives and have a higher employment rate have been reported in previous studies [51, 52]. These highlight that an active community presence and long-established relationships could provide support and promote help-seeking [20, 53]. The significant association between the mode of onset of psychosis and DUP is in line with previous research [11, 54] suggesting that the speed at which psychosis develops influences help-seeking and treatment delays. That is, people with FEP who experience an acute onset of psychosis, and a sudden change in their behaviour, may be more likely to seek help quicker or have help sought for them [27, 55].

In contrast to some previous studies [9, 11, 41], we found no evidence of an association between DUP and sociodemographic variables. The lack of differences in DUP by sociodemographic variables in the present study might be explained by our relatively low power. For example, we observed a longer DUP in the unemployed (161.5 days) compared with employed (74.0 days) patients. Our overall median DUP was 129 (IQR: 27.5–542.0) days, longer than those observed in urban settings, by Oduola et al. ((median DUP 93 [IQR 19–447] days) [9]; Morgan et al. (median DUP 9 [IQR:2–40] weeks) [11], and Kirkbride et al. (median DUP 69.5 [IQR:22.5–314.0] days) [6]. However, these previous studies had large sample sizes and were able to detect relationships between DUP and sociodemographic characteristics. Meanwhile, our observation of a long median DUP among patients from rural areas and the associations between DUP and insidious mode of onset are in keeping with other international studies. In a Japanese study of a similar FEP sample (*n* = 108) in a rural area, Lihong et al. [56] reported a long median DUP of 10.5 (IQR: 0.1–312) months. They found that a longer DUP is associated with an insidious mode of onset [56]. This is also echoed in a more recent study from Nepal, where Limbu and colleagues (2024) reported a median DUP of 3.0 (IQR 23.5) months in a sample of 86 patients from rural areas. They also found a strong association between DUP and an insidious most of onset [57].

Our findings of an association between living circumstances, employment status and mode of onset of psychosis have been reported in other studies [9, 25]. Furthermore, our observation of a correlation between an acute onset and living with family/relatives and being in employment points to the importance of social networks and significant others (e.g., co-workers) being able to recognise psychotic symptoms or changes in behaviour. Hence, they could facilitate help-seeking [25, 27, 58]. The link between DUP and mode of onset of psychosis is notable, particularly given the latter assesses the speed at which psychotic symptoms develop. Our finding that a gradual onset of psychosis was more common among unemployed patients points to the role of social isolation and reduced social functioning, suggesting difficulties in identifying psychotic symptoms [11, 59]. Therefore, recognising and detecting the speed of psychosis are more important as part of efforts to reduce treatment delays. Indeed, initiatives and interventions aimed at the general public and non-healthcare professionals are promising for early detection, better access to treatment and reducing DUP [60–62].

### Implications for clinical practice

The evidence of associations between a gradual and insidious onset of psychosis and DUP highlights the need for strategies to recognise less noticeable symptoms of psychosis (e.g., social isolation or withdrawal). For example, some individuals at the early stages of illness may present with symptoms of lesser severity and duration or non-psychotic symptoms such as anxiety and depression [63]. It is, therefore, important that EIS pay attention to the thresholds and boundaries of the criteria used for assessing first episode psychosis. For some EIS, the threshold is quite strict [64]. For instance, in some services, the thresholds of positive scale on the Positive and Negative Syndrome Scale (PANSS) need to be met, [65], meaning patients who do not meet their screening criteria (e.g., clear-cut psychotic symptoms) may be directed elsewhere for treatment. However, such criteria will only prolong the DUP. Hence, pragmatic screening criteria that are sensitive to the prodromal context of the early development of psychosis are needed when assessing FEP patients. We acknowledge that our study was conducted before the introduction of new Access and Waiting Time Standards (AWTS) for EIS in England, UK in 2016 [64], which outlined that people referred for FEP should receive treatment from an EIS within two weeks and extended the upper age limit from 35 years to 65 years; hence potentially reducing treatment delays. However, only a few studies have been conducted since the implementation of the AWTS. Most of the available research has evaluated the policy’s implementation, estimating the proportion of people seen within two weeks [66–68]. Since the AWTS were implemented, there have yet to be studies specifically examining DUP and associated characteristics, particularly rurality.

### Future research

More research is needed on DUP and pathways to care in rural populations. Studies with larger samples exploring DUP and duration of untreated illness in at-risk-mental-state populations and examining other clinical variables such as symptom severity and positive vs. negative symptoms, are also warranted.

## Conclusions

Our results suggest that the mode of onset of psychosis is an important indicator of treatment delay. Sociodemographic variations in FEP exist in rural populations, and our findings are similar to those observed in urban settings. The results could inform service planning and delivery. Addressing mental health in rural communities is a complex issue that requires a multi-faceted approach. Increasing awareness about psychosis and recognising its symptoms, as well as addressing barriers to treatment, may help to reduce DUP in rural areas.

## Electronic supplementary material

Below is the link to the electronic supplementary material.

Supplementary Material 1

Supplementary Material 2

## Declarations

### Data sharing

No additional data are available.
