# Supplementary material for: Associations between rural/urban status, duration of untreated psychosis and mode of onset of psychosis: a mental health electronic clinical records analysis in the East of England, UK
Source: Soc Psychiatry Psychiatr Epidemiol. 2024 Sep 9;60(6):1323–34. doi: 10.1007/s00127-024-02758-3 (PMC12162678; doi:10.1007/s00127-024-02758-3)
Supplement: Supplementary file 2 — Supplementary Material 2 [file 127_2024_2758_MOESM2_ESM.docx]

Supplementary Material 2: SQL Syntax

Project title:

Associations between rural/urban status, duration of untreated psychosis and mode of onset of psychosis: a mental health electronic clinical records analysis in the East of England, UK. Sheri Oduola and Karolina Kaminska.

Date: 2013-05-01 to 2015-04-30.

All patients accepted to CAMEO during this period.

Output:

Duration of untreated psychosis

-Will be calculated using the date of contact with mental health services and the date of onset of psychosis

Mode of onset of psychosis.

-Will be identified through when symptoms start (days, weeks, months)

Duration and Mode results refined by searching for terms like:

-psychosis

-delusions

-first episode of psychosis

-voices

-hallucinations

-onset of symptoms.

Structured data regarding service use:

-date of acceptance to CAMEO

-date of admission to inpatient

-date of discharge.

-date of acceptance to other CPFT service, if CAMEO was not first service contacted.

Sociodemographic data:

-age

-gender

-ethnicity

-living circumstances

-employment status

-level of education

-marital status (possibly)

-LSOA and IMD scores closest to date accepted to CAMEO

***/

--Search 1. Define the cohort. Everybody should be included here.

--Can change the date range easily to get more than one year.

SELECT *

FROM Workspace.dbo.CAMEO_Refs_KK_2yr

ORDER BY Cohort_n, rid

--Search 2. Diagnoses for the cohort

SELECT rid, diagnosis, Diagnosis_Code, Diagnosis_Start_Date, Diagnosis_End_Date, Entered_By_Main_Specialty_Description

FROM RiO.dbo.diagnosis

WHERE rid in (SELECT rid FROM Workspace.dbo.CAMEO_Refs_KK_1yr)

--Search 3. Addresses

SELECT rid, Address_From_Date, Address_To_Date, Address_Type_Description, Primary_Care_Group_Description,

bua11, buasd11, casward, imd, lea, lsoa01, lsoa11, msoa01, msoa11, nuts, oac01, oac11, parish, pcon, pct, ru11ind,

statsward, ur01ind

FROM rio.dbo.Client_Address_History

WHERE rid in (SELECT rid FROM Workspace.dbo.CAMEO_Refs_KK_1yr)

--Search 4. Inpatient stays

SELECT rid, Admission_Date, Admission_Method_Description, Admission_Source_Description, Client_Classification_Description,

Discharge_Date, Discharge_Comment, Discharge_Destination_Description, legal_status_description,

referral_source_description, referrer

FROM RiO.dbo.Inpatient_Stay

WHERE rid in (SELECT rid FROM Workspace.dbo.CAMEO_Refs_KK_1yr)

--Search 5. Searching patient text for the occurrence of a word or phrase

--Enter your word or phrase in both places

SELECT DISTINCT rid,tablename, columnname, docdate, datum,

substring (Datum, (charindex( 'discharge', Datum) -50), 400 ) as String --<<--- Change numbers here for a longer string

FROM crateutils.dbo.Combined_Text

WHERE Datum like '%discharge%'

AND rid = **********************

--AND rid in (SELECT rid FROM Workspace.dbo.CAMEO_Refs_KK_1yr)

--Search 6. To look at the whole document identified in search 5.

--Note that the date field will be called something different in each table, run first 2 lines to find out what!!

SELECT *

FROM RiO.dbo.Clinical_Documents --<<--- from tablename column

WHERE rid = ********************** --<<--- from rid column

AND Date_Created = '2013-10-30 10:19:15.340' --<<--- from docdate

--OR

--Search 6b. The full text is in the Datum field

SELECT *

FROM CrateUtils.dbo.Combined_Text

WHERE rid = ********************** --<<--- from rid column

--AND docdate = '2014-08-27 09:57:19.750'

ORDER BY TableName, docdate

-- 2021-01-20

-- Additional searches

SELECT cam.*, start_CAMEO, Address_From_Date, Address_To_Date, address_type_description, lsoa01, casward --more geographical identifiers available

FROM rio.dbo.client_address_history cah

INNER JOIN Workspace.dbo.CAMEO_Refs_KK_2yr cam

ON cah.rid = cam.rid

WHERE start_CAMEO BETWEEN address_from_date AND ISNULL(Address_to_date, GETDATE())

-- On search #6b, please can we have this split into 3 group, i.e.

-- (a) Core Assessment (social, medical, family, forensic, mental state, presenting problems, summary plan etc),

-- (b) clinical documents- progress note, discharge letters, referral letter etc.

-- (c) Referral/acceptance i.e. inpatient admission date, referral date, assessment date by any CPFT service, referral reason.

-- New search 6b (a) Core Assessment (social, medical, family, forensic, mental state, presenting problems, summary plan etc)

-- New search 6b (b) Clinical documents (progress note, discharge letters, referral letter etc.)

SELECT *

FROM CrateUtils.dbo.Combined_Text

WHERE rid IN (SELECT rid FROM Workspace.dbo.CAMEO_Refs_KK_2yr) --<<--- for all the patients at once

-- WHERE rid = **********************--<<--- from rid column

AND tablename LIKE 'CPFT_CORE%'

-- AND tablename IN ('Progress_notes', 'Clinical_Documents', 'UserAssessipdischargesum1')

ORDER BY rid, TableName, docdate

-- New search (c) Referral/acceptance i.e. inpatient admission date, referral date, assessment date by any CPFT service, referral reason.

SELECT rid, referral_datetime, discharge_datetime, discharge_comment, discharge_reason, patient_area_description,

referrer, Specialty_Referred_To_Description, Team_Referred_To_Classification_Group_Description,

Team_Referred_To_Description, Urgency_Description

FROM rio.dbo.referral

WHERE rid IN (SELECT rid FROM Workspace.dbo.CAMEO_Refs_KK_2yr)

ORDER BY rid, Referral_DateTime
